# P-235. Fracture and Musculoskeletal Injury Risk in Active Duty Military with HIV Compared to Those without HIV: A Retrospective Virtual Cohort Study

**DOI:** 10.1093/ofid/ofaf695.457

**Published:** 2026-01-11

**Authors:** Jacqueline M Causbie, Mackensie Horn, Hsing-Chuan Hsieh, Senay Topal, Anuradha Ganesan, Robert O’Connell, Brian Agan, Brenna M Roth

**Affiliations:** Walter Reed National Military Medical Center; Infectious Disease Clinical Research Program, Bethesda, Maryland; Infectious Disease Clinical Research Program, Bethesda, Maryland; Uniformed Services University of the Health Sciences, North Bethesda, MD; Uniformed Services University of Health Sciences, Bethesda, Maryland; Infectious Disease Clinical Research Program, USUHS, Bethesda, Maryland; Infectious Disease Clinical Research Program, Department of Preventive Medicine and Biostatistics, Uniformed Services University of the Health Sciences, Bethesda, MD, USA, Bethesda, Maryland; Henry M. Jackson Foundation for the Advancement of Military Medicine, Baltimore, Maryland

## Abstract

**Background:**

HIV infection is associated with decreased bone mineral density and increased fracture risk. Fractures are a noted cause of morbidity among active duty service members (ADSM), often due to high-impact physical activity. There is little available literature on fracture risk in service members with HIV. This study evaluated differences in risk of fractures and other musculoskeletal injuries (MSKI) in a matched cohort of service members with and without HIV (PWH, PWoH) using retrospective data from the DoD HIV Virtual Cohort Study (VCS).

**Figure:**
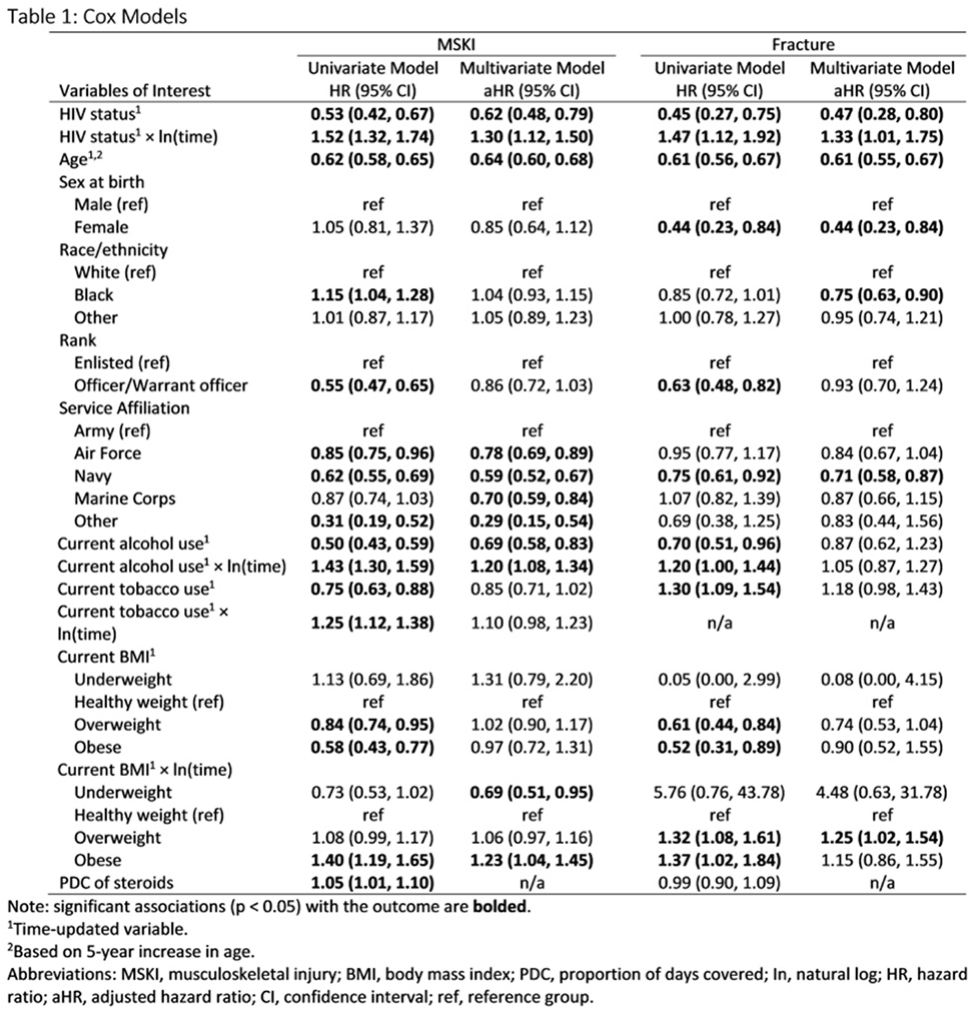

**Figure:**
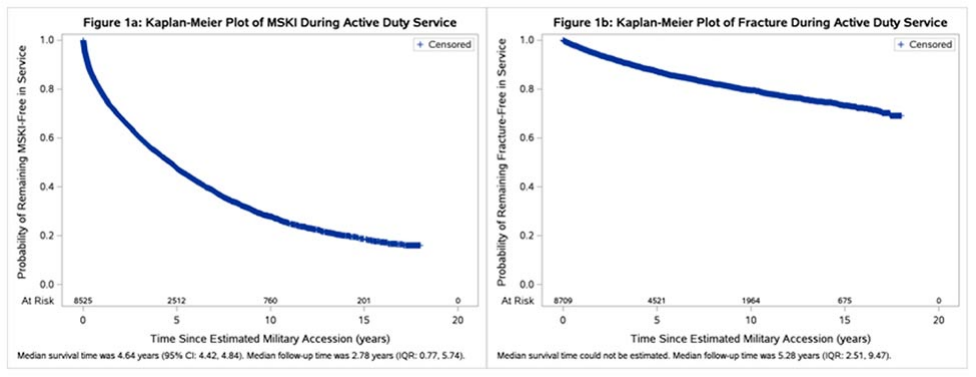

**Methods:**

De-identified data were gathered from VCS participants who had at least one medical encounter while AD between 2001 and 2018. Kaplan-Meier survival curves were plotted for static risk factors. Multivariable Cox proportional hazard models with time-updated HIV status were used to examine risk factors for fractures and other MSKI. If the assumption of proportional hazards was violated, then an interaction term of the covariate and time was added to the model to address the time-varying effect of the covariate.

**Figure:**
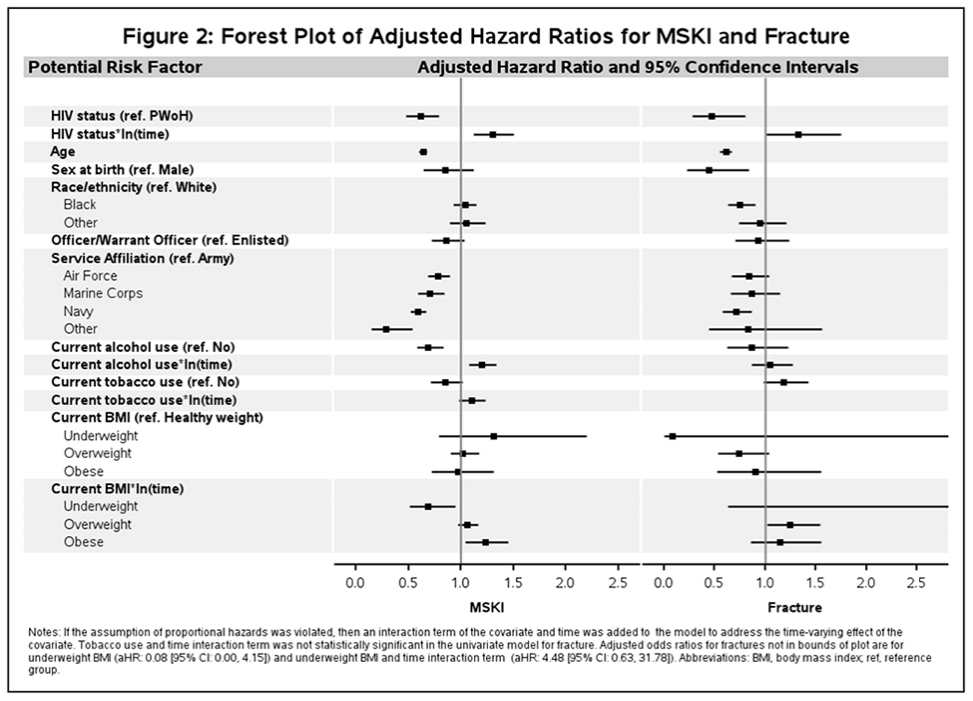

**Results:**

We analyzed 8742 ADSM (96% male, 48% Black, 26% PWH). Median time from military accession to HIV diagnosis was 3.41 years (interquartile range (IQR) 1.54-6.17). There were 1335 fractures (132 among PWH) and 4849 MSKI (364 among PWH). PWH were less likely than PWoH to have experienced fracture (adjusted hazard ratio (aHR) 0.47) or other MSKI (aHR 0.62) initially. However, the initial protective effect diminished over time for both fractures and MSKI (*time aHR 1.33 and 1.30). Service in the Army was associated with a higher risk of fracture compared to the Navy and a higher risk of MSKI compared to all branches. Increasing age was associated with lower risk of fracture (aHR 0.61) and MSKI (aHR 0.64).

**Conclusion:**

PWH were at lower risk of fracture and other MSKI initially compared to PWoH, but this protective effect diminished over time. Increasing age was associated with lower risk of MSKI, possibly as the result of decreasing risk due to differing jobs by age. Risk of MSKI varied among branches with the highest risk in the Army. Future research should investigate the role of military occupation and unit assignment, as this is affected by both age and HIV status and plays a role in determining a service member’s amount of high-impact activity.

**Disclosures:**

All Authors: No reported disclosures

